
JOURNAL INFORMATION
==============================
NLM Title Abbreviation: ScientificWorldJournal
Journal ID: ScientificWorldJournal
NLM Journal ID: 101131163
Journal ID: TSWJ

The Scientific World Journal

ISSN: 2356-6140
EISSN: 1537-744X
Publisher: Wiley

ARTICLE INFORMATION
==============================
PMCID: PMC6009359
PMID: 29973838
DOI: 10.1100/tsw.2002.62
Article ID: 396814
Article version: 1
Subjects: Short Report

Protein Misfolding and Human Disease

Dobson Christopher
Department of Chemistry, University of Cambridge, Lensfield Road, Cambridge CB2 1EW, UK
Publication date: 2002
Electronic publication date: 2002 Jun 26
Volume: 2
First page: 132
Last page: 132
Copyright: Copyright © 2002 Christopher Dobson.
Copyright year: 2002
License: This is an open access article distributed under the Creative Commons Attribution License, which permits unrestricted use, distribution, and reproduction in any medium, provided the original work is properly cited.
License URL: https://creativecommons.org/licenses/by/3.0/

==============================
Advances in Gene Technology: The Genome and Beyond –
Structural Biology for Medicine (Proceedings of the 2002 Miami
*Nature Biotechnology* Winter Symposium)
*TheScientificWorld* 2002, 2(S2), 132
ISSN 1532-2246; DOI 10.1100/tsw.2002.62

### PROTEIN MISFOLDING AND HUMAN DISEASE

Christopher M. Dobson
Department of Chemistry, University of Cambridge, Lensfield Road, Cambridge CB2 1EW, U.K.

Protein folding is perhaps the most fundamental process associated with the generation of functional structures in biology. There has been considerable progress in the last few years in understanding the underlying principles that govern this highly complex process. Central to much of this progress has been the development of ideas as to the nature of the energy surface or landscape for a folding reaction. These ideas have arisen from a combination of theoretical analysis and experimental investigation[1]. Of particular importance in the latter has been the concerted application of a wide range of experimental techniques, each able to describe aspects of the structural changes taking place during the folding process. NMR spectroscopy and protein engineering have both been key methods in this approach because of their ability to provide structural and dynamical information at the level of individual residues. Recently, new approaches have been devised that combine experimental data directly with simulation techniques to define the structures of key species on the folding surface[2].

Recently, much research has also focused on the realisation that proteins can misfold *in vivo* and that this phenomenon is linked with a wide range of diseases, particularly those associated with modern highly developed societies. We have been investigating in particular the nature of the amyloidogenic diseases (that include Alzheimer's disease and the spongiform encephalopathies, e.g., BSE and CJD) in which protein misfolding leads to the aggregation of proteins, often into fibrillar or thread-like structures. We have looked in particular at a nonneuropathic amyloidosis associated with lysozyme, one of our model systems for investigating the fundamentals of protein folding[3]. This work has recently been extended by the finding that many proteins not asociated with clinical manifestations of disease can form amyloid structures in the laboratory under appropriately chosen conditions[4,5]. Such findings have led us to put forward ideas as to a common origin of the various diseases associated with the formation of amyloid structures. We have also speculated more generally that the avoidance of aggregation could be a significant driving force in the evolution of protein sequences and structures[6].

### REFERENCES

1. Dinner, A.R. et al. (2000) Trends Biochem. Sci. 25, 331–339.
2. Vendruscolo, M. et al. (2001) Nature 409, 641–645.
3. Booth, D.R. et al. (1997) Nature 385, 787–793.
4. Chiti, F. et al. (1999) Proc. Natl. Acad. Sci. U. S. A. 96, 3590–3594.
5. Fandrich, M. et al. (2001) Nature 410, 165–166.
6. Dobson, C. (2001) Philos. Trans. R. Soc. London Ser. B 356, 133–145.
